# Physical activity for weight loss maintenance in people with and without physical disability: The international weight control registry

**DOI:** 10.1016/j.dhjo.2026.102024

**Published:** 2026-01-05

**Authors:** Julianne G. Clina, R. Drew Sayer, James E. Friedman, Tsz Kiu Chui, Anna M. Gorczyca, Sai Krupa Das, L. Adele Fowler, Susan B. Roberts, James O. Hill

**Affiliations:** a University of Kansas Medical Center, Department of Internal Medicine, United States; b University of Alabama at Birmingham, Department of Family and Community Medicine, United States; c University of Alabama at Birmingham, Department of Nutrition Sciences, United States; d Tufts University, Jean Mayer USA Human Nutrition Research Center on Aging, United States; e Dartmouth College, Geisel School of Medicine, United States

**Keywords:** Obesity, Physical activity, Physical disability, Weight management

## Abstract

**Background:**

People with physical disabilities (PWD) are at increased risk for obesity compared to people without disability (PWoD). PWD face barriers to physical activity (PA), which is important for weight loss maintenance (WLM) in PWoD. However, the role of PA in WLM has not been evaluated in PWD.

**Objective:**

The purpose of this study was to examine differences in PA between PWD and PWoD and whether PA is associated with WLM success among PWD.

**Methods:**

Data were derived from 536 participants already enrolled in the International Weight Control Registry, which included targeted recruitment of PWD. Physical disability was self-reported via study-designed survey capturing presence and type of disability. PA was assessed using the International Physical Activity Questionnaire, and weight history was self-reported as “Successful” (lost weight and maintained ≥1 year), “Regain,” or (Lost but regained within 1 year). PA was compared between PWD and PWoD and among PWD by weight history status using generalized regression models.

**Results:**

PWD (n = 174) reported more sedentary time (β = 0.34; 95 % CI = 0.16–0.52, p < 0.001), and greater odds of reporting zero total physical activity (OR = 5.18, 95 % CI = 2.19–12.24, *p* < 0.001), zero total leisure physical activity (OR = 2.22, 95 % CI = 1.51–3.26, *p* < 0.001), and zero leisure vigorous physical activity (OR = 1.87, 95 % CI = 1.15–3.02, *p* = 0.011). There were no significant differences in PA among PWD based on weight history status.

**Conclusions:**

PA may not be a critical factor influencing weight maintenance in PWD. Even among successful participants, PWD report lower PA than PWoD, highlighting the need for inclusive PA programs.

## 1. Introduction

Currently, approximately 13 % of Americans have a physical disability or condition that limits mobility.^1^ The number of people living with disabilities continues to increase, largely due to the increase in life expectancy in the last several decades.^1,2^ People with physical disability (PWD) face higher risk for several negative health outcomes when compared to people without physical disabilities (PWoD).^3^ For example, prevalence of obesity is higher in populations with physical disability., with Townsend et al. reporting an obesity prevalence of 40.1 % in PWD versus 30.5 % in PWoD.^4^ In PWD, obesity has been associated with further declines in mobility and decreased health-related quality of life overall,^5^ demonstrating the importance of weight management for this population. While several studies have investigated weight loss in PWD,^6-8^ little is known about weight loss maintenance in this population. In general, studies have concluded that following weight loss, most participants regain all or most of the weight lost within one year,^9,10^ thus identifying factors associated with greater weight loss maintenance for PWD is important for long term health outcomes.

In studies including PWoD, regular physical activity has been associated with long-term weight loss maintenance,^11-13^ with evidence suggesting physical activity engagement greater than the Physical Activity Guidelines for Americans of 150 min of moderate activity per week may be beneficial for weight loss and maintenance.^14,15^ However, PWD face environmental barriers to regular physical activity participation including a lack of transportation access, and a lack of accessible exercise equipment and facilities.^16,17^ In addition, personal factors such as lower socioeconomic status and varying levels of functional limitations may also influence physical activity participation.^16,17^ It has consistently been reported that PWD engage in less physical activity than people without disability (PWoD).^18,19^ However, it is unclear how this may impact weight management efforts in PWD.

The International Weight Control Registry (IWCR) is a longitudinal study designed to capture information regarding weight management strategies including weight history data, and behavioral, environmental, psychological, and economic contributors to weight management.^20^ The IWCR includes participants who were defined as “Successful”, defined as having lost weight and maintained weight loss for at least 1 year or “Regain”, those who successfully lost weight but regained the lost weight within 1 year. Recently the IWCR was modified to include PWD, and PWD were specifically recruited to participate in the study. The objectives for this evaluation were to compare physical activity engagement of PWD and PWoD in individuals with a history of weight loss, and compare physical activity engagement of PWD in those who maintained versus regained weight. It was hypothesized that 1) PWD will generally participate in less physical activity than PWoD, regardless of weight history category and 2) PWD who successfully maintain weight loss will participate in more physical activity than those who regain weight.

## 2. Methods

### 2.1. Participants

**Fig. 1.**
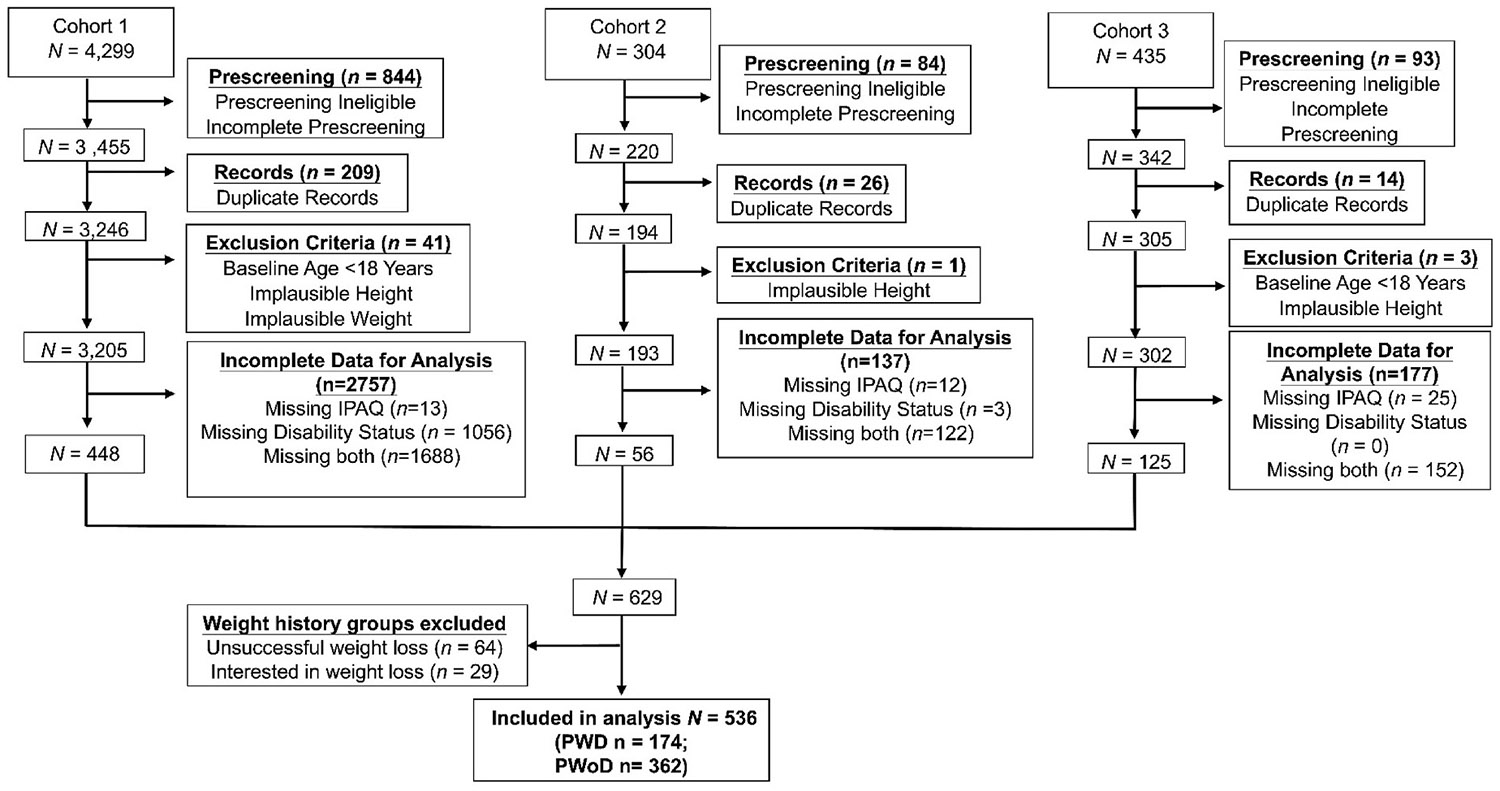
CONSORT Diagram Abbreviations: IPAQ, International Physical Activity Questionnaire; PWD, people with physical disability; PWoD; people without physical disability.

A diagram depicting participant enrollment is presented in Fig. 1. All methods were approved by each University's Institutional Review Boards and all participants completed a consent process prior to enrolling in the study. As described in previous reports, participants also self-identified as one of four weight loss categories upon study entry: (1) Successful, those who are successful at losing weight and successful at maintaining lost weight for at least 1 year, (2) Regain, those who successfully lost weight but regained the weight they lost, (3) Unsuccessful, those who were not able to lose weight, or (4) Interested, those attempting weight loss for the first time.^21^ For the purpose of these analyses, only participants in the “Successful” or “Regain” weight history groups were included due to small sample sizes present in the other two categories. Data from three cohorts were included in these analyses. Cohort 1 was recruited from December 2020–October 2021; Cohort 2 from June 2023–August 2023; and Cohort 3 from March 2023–August 2023. Recruitment for Cohort 1 occurred prior to the inclusion of questions on physical disability status in the IWCR battery; for these participants, disability status was assessed with a follow-up questionnaire sent in March 2023. Participants from Cohorts 2 and 3 were recruited following implementation of the disability status questions into the IWCR battery and assessed for disability status with the baseline questionnaires. Participants from all three cohorts were recruited using a variety of methods that included contacting participants from previous research studies, research institute recruitment databases, affiliated university hospital networks, social media, community engagement, and United States Department of Agriculture extension partnerships. Cohort 3 was a specific recruitment effort to include more PWD in the study sample and used similar recruitment methods as the other cohorts while targeting past research studies, research institute recruitment databases, and community partnerships that included PWD specifically.

### 2.2. Sociodemographic information

Demographic information (age, sex, race, ethnicity, income, etc.) and weight history (current BMI and weight loss category) were self-reported by participants. Disability status and use of assistive devices were assessed in a survey designed by the research team, based on surveys used for enrollment into programs from the National Center on Health, Physical Activity and Disability (NCHPAD).^22^ Participants were asked “Do you have a physical disability or any condition that limits your mobility” and if “Yes” was selected, participants were prompted to select the type of physical disability, and asked “Do you use any assistive devices for ambulation”. Individuals were allowed to select multiple disabilities and multiple assistive devices used for ambulation as appropriate. Listed disabilities to select from included amputation, paralysis, osteoarthritis, rheumatoid arthritis, myasthenia gravis, edema, Friedreich ataxia, cerebral palsy, multiple sclerosis, spinal cord injury, spina bifida, blindness, peripheral neuropathy, diabetic retinopathy, Parkinson's disease, traumatic brain injury, stroke, muscular dystrophy, macular degeneration, postural orthostatic tachycardia syndrome, joint pain, and other, and assistive devices included cane, walker, crutches, rollator, push wheelchair, power wheelchair, guide dog, white cane, rollator, upper or lower limb prosthetic, reacher tool, ankle-foot orthoses, or other.

### 2.3. Physical activity survey

Physical activity was assessed using the International Physical Activity Questionnaire (IPAQ), which collects data on physical activity engagement across five domains including work, household, leisure, transportation, and sedentary time.^23-25^ Metabolic Equivalent Task (MET) minutes per week were calculated using the established data cleaning procedures and scoring guidelines.^26^ Activities lasting less than 10 min were recoded as 0 min. Reported activity times (walking, moderate, vigorous) over 180 min per day were truncated to 180 min per day. Total physical activity time was the sum of all truncated activity times across five domains. Total physical activity time exceeding 960 min per day were excluded. MET minutes per week were calculated by multiplying activity minutes per week by MET values: 3.3 for walking, 4.0 for moderate, and 8.0 for vigorous activity. Leisure-time MET minutes per week were calculated similarly for walking, moderate, and vigorous activities. Total leisure-time MET minutes per week were summed across MET minutes per week for walking, moderate, and vigorous leisure activities. Weekly sedentary time was calculated by summing reported sitting time on weekdays and weekends. Cohort 3 participants completed a version of the IPAQ modified to include PWD.^24,25^ This adapted survey was designed to be used in both PWD and PWoD and has demonstrated acceptable test-retest reliability (Intraclass correlation coefficients (ICC): 0.690; 95 % CI, 0.581–0.770) convergent validity with the original instrument (ICC: 0.727; 95 % CI, 0.579–0.829; P < 0.001), and construct validity when compared with accelerometry (ICC: 0.406; 95 % CI, 0.166–0.596; P = 0.001).^25^ Changes to the survey primarily included providing additional instructions for wheelchair users.^24,25^ Participants in Cohorts 2 and 3 were asked about presence of physical disability or mobility limitation during the baseline IWCR questionnaire packet, however as described earlier, this was not a part of data collection during cohort 1. Participants in Cohort 1 were sent the same disability assessment as Cohorts 2 and 3 via email during the first 1 year follow up assessment, as a part of the ongoing longitudinal data collection for the IWCR.

All study data were collected and managed using REDCap electronic data capture tools hosted at the University of Alabama at Birmingham.^27,28^ REDCap is a secure, web-based software platform designed to support data capture for research studies, providing (1) an intuitive interface for validated data capture; (2) audit trails for tracking data manipulation and export procedures; (3) automated export procedures for seamless data downloads to common statistical packages; and (4) procedures for data integration and interoperability with external sources.

All analyses were completed using R version 4.5.1 (R Core Team, 2024),^29^ and all hypotheses were two-sided with α = 0.05. Model estimates were generated with the *broom* and *broom. mixed* packages. Data processing was performed with the *tidyverse* package, and examined prior to analysis using the *DescTools* package. For analysis, participants were grouped by disability status (PWD vs people without disability), self-reported weight history category (successful vs regain), and within PWD, assistive device use (yes/no). Baseline characteristics of participants were compared between disability status groups using Chi-squared tests for categorical data or Wilcoxon rank-sum tests for continuous data. Distributions for physical disabilities and assistive device use among PWD were assessed using descriptive statistics.

We used generalized regression models to evaluate associations for each domain of physical activity with the following primary predictors: physical disability status, self-reported weight history category, and assistive device use within PWD. Additional subgroup analyses were conducted within weight history categories. All associations were adjusted for baseline age (in years). Associations with sedentary time were evaluated with generalized linear models fitted to a Gaussian distribution. For all other domains of physical activity, distributions were highly skewed with a large proportion of zeros; additionally, zeros for moderate and vigorous leisure time physical activity arose from two distinct mechanisms: (1) participants reporting no total leisure-time physical activity (total leisure physical activty = 0), and (2) participants reporting some leisure activity but none at moderate intensity. A standard negative binomial model would not adequately account for this excess zero inflation, and a Poisson model was inappropriate due to overdispersion. Therefore, associations for the remaining domains of physical activity were evaluated with zero-inflated negative binomial (ZINB) models, which combine a negative binomial count process for non-zero observations with a separate logit model for the probability of structural zeros.^30^ This approach allows for more accurate estimation of associations while accounting for heterogeneity in zero-generating processes. ZINB models were fitted with the *glmmTMB* package in R and specified via two components:

#### Count component (negative binomial):

included the primary predictor and baseline age to model expected MET-minutes among participants engaging in moderate activity.

#### Zero-inflation component (logit):

included the primary predictor and modeled probability of zero physical activity for the outcome. In models for moderate and vigorous leisure-time physical activity, an indicator variable was also included that specified whether total leisure-time physical activity was zero.

All ZINB models were fitted using the “nbinom1” family (variance proportional to the mean). Model diagnostics included simulation-based residual checks (uniformity, dispersion, zero-inflation, and outlier tests), Pearson dispersion statistics, and predictive calibration plots using the *DHARMa* package. Influence diagnostics (Cook's distance) were also examined. Estimates for the count component were expressed as incidence rate ratios (IRRs), and for the zero-inflation component as odds ratios (ORs), with 95 % confidence intervals derived from Wald approximations.

## 3. Results

A total of 536 participants were included in this analysis. Of these participants, 174 (32.5 %) reported having a physical disability or condition that limits mobility. Table 1 presents demographic characteristics of the sample by disability status. PWD and PWoD had similar distributions by race and ethnicity, and the majority of participants in both groups were female (PWD, 81.6 %; PWoD, 80.9 %), white race (PWD, 76.4 %; PWoD, 81.2 %), and non-Hispanic ethnicity (PWD, 90.2 %; PWoD 93.1 %). Compared to PWoD, participants in the PWD group were older (57.4 ± 11.4 vs 52.6 ± 14.1, *p* < 0.001), had a higher BMI (35.9 ± 10.5 vs 31.1 ± 7.6, *p* < 0.001), and were more likely to have lower income and less likely to have full-time employment.

Table 2 presents presence/types of physical disability and assistive device use among PWD participants. The most commonly-reported disabilities or conditions that limit mobility were joint pain (72.4 %), osteoarthritis (50.6 %), edema (16.7 %), peripheral neuropathy (15.5 %), multiple sclerosis (13.8 %) and spinal cord injury (12.1 %). Of the 174 PWD, 70 (40.2 %) reported use of assistive devices; in these participants, the most commonly used devices were canes (64.3 %), wheelchairs (42.9 %), and walkers (30.0 %).

**Fig. 2.**
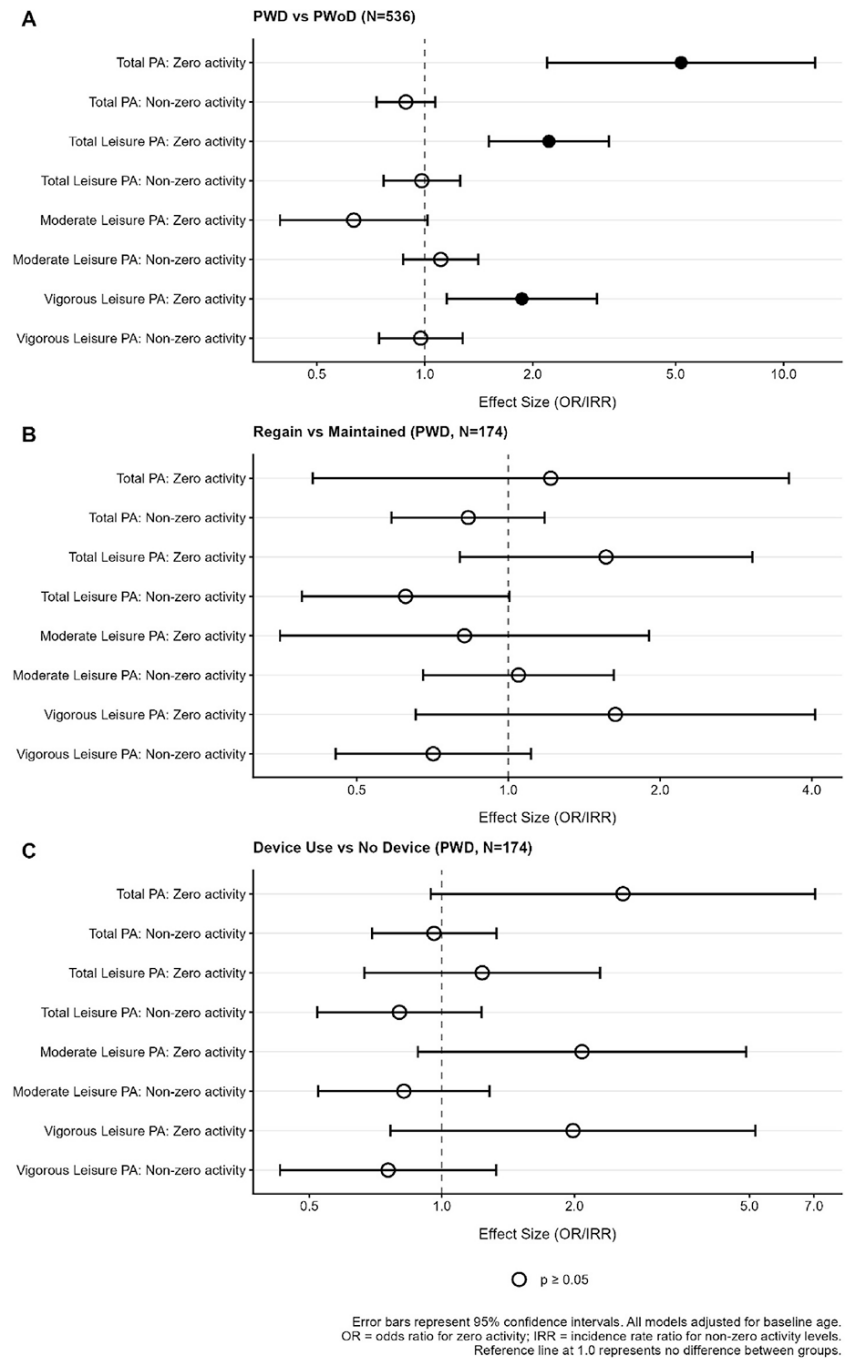
Physical Activity Comparisons by Group A. Notes: Persons with Disability vs Persons without Disability (Reference), N = 536. Error bars represent 95 % confidence intervals. All models adjusted for age. Effect Size (OR/IRR for Physical Activity)Abbreviations: IRR = incidence rate ratio; OR = odds ratio; PA = Physical Activity; PWD, = People with Disability; PWoD = People without Disability. B. Note: Persons with Disability: Regain vs Successful Groups (Reference), N = 174. Error bars represent 95 % confidence intervals. All models adjusted for age. Effect Size (OR/IRR for Physical Activity)", Abbreviations: IRR = incidence rate ratio; OR = odds ratio; PA = Physical Activity. C. Note: Persons Using vs. Not Using Assistive Devices (Reference), N = 174. Error bars represent 95 % confidence intervals. All models adjusted for age. Effect Size (OR/IRR for Physical Activity). Abbreviations: IRR = incidence rate ratio; OR = odds ratio; PA = Physical Activity.

Results from zero-inflated negative binomial models comparing physical activity outcomes between PWD and PWoD in Fig. 2A. PWD reported more sedentary time than PWoD (β = 0.337; 95 % CI 0.155–0.520; *p* < 0.001). PWD also had higher odds of reporting zero total physical activity (OR: 5.18; 95 % CI = 2.19–12.38, p < 0.001), total leisure physical activity (OR: 2.22; 95 % CI = 1.51–3.26, p < 0.001), and vigorous leisure physical activity (OR: 1.87; 95 % CI = 1.15–3.02, p = 0.011). There was no difference in PWD when comparing odds of reporting zero moderate leisure physical activity (OR: 0.63; 95 % CI = 0.40–1.02, p = 0.59). Among participants with non-zero values, disability status was not significantly associated with outcome rates. Rate ratios were 0.89 (95 % CI = 0.73–1.07, p = 0.21) for total physical activity, 0.98 (95 % CI = 0.77–1.25, p = 0.88) for total leisure physical activity, 1.11 (95 % CI = 0.87–1.41, p = 0.41) for Moderate Leasure Physical Activity, and 0.94 (95 % CI = 0.75–1.27, p = 0.85) Vigorous Leisure Physical Activity.

Results from models comparing physical activity outcomes among PWD by weight history category are presented in Fig. 2B. Weight history category was not significantly associated with the odds of reporting zero outcomes for any measure. Specifically, the odds ratios were: Total physical activity: 1.21 (95 % CI = 0.41–3.60, p = 0.73), total leisure physical activity: 1.56 (95 % CI = 0.80–3.05, p = 0.19), moderate leisure physical activity: 0.82 (95 % CI = 0.35–1.90, p = 0.64), and vigorous leisure physical activity: 1.63 (95 % CI = 0.65–4.06, p = 0.29). Among participants with non-zero values, the weight history category was also not significantly associated with outcome rates. Rate ratios were 0.83 (95 % CI = 0.59–1.18, p = 0.30) for total physical activity, 0.63 (95 % CI = 0.39–1.00, p = 0.052) for total leisure physical activity, 1.05 (95 % CI = 0.68–1.62, p = 0.84) for moderate leisure physical activity, and 0.71 (95 % CI = 0.45–1.11, p = 0.13) Vigorous Leisure Physical Activity. Finally, there were no differences in sedentary time based on weight history category (β = 0.282; 95 % CI −0.38 to −0.07; *p*=0.09).

Table 3 presents comparisons of physical activity between PWD and PWoD among participants in the “Successful” group. Among those “Successful”, PWD had significantly more sedentary time (β = 0.48; 95 % CI = 0.15–0.80; *p* = 0.004) and greater odds of reporting zero total leisure physical activity (OR = 4.25, 95 % CI = 1.99–9.05, *p* < 0.001) compared with PWoD. For total physical activity score, the zero-inflated logistic regression model did not converge; among those reporting nonzero activity, there were no significant differences in activity levels between PWD and PWoD (IRR = 0.94, 95 % CI = 0.73–1.19, p = 0.593). No differences between groups were observed for total leisure activity, moderate leisure activity, or vigorous leisure activity.

Table 4 presents comparisons of physical activity between PWD and PWoD among participants in the 'Regain' group. PWD had significantly more sedentary time (β = 0.35, 95 % CI = 0.125–0.575, p = 0.002) and were more likely to report zero total physical activity (OR = 3.21, 95 % CI = 1.28–8.05, p = 0.013). No differences in reported activity levels among those with non-zero activity.

Finally, comparisons of physical activity engagement by assistive device use among PWD are shown in Fig. 2C. Those who used assistive devices reported more sedentary time (β = 0.35, 95 % CI 0.05–0.65; *p* = 0.021). However, there were no significant differences in any of the other physical activity domains based on assistive device use.

## 4. Discussion

The purpose of this investigation was to leverage an online weight management registry to compare physical activity engagement based on disability status and weight history. This analysis found that PWD report less physical activity across several domains, and more sedentary time than PWoD. Contrary to the hypothesis, no significant differences were observed among PWD based on weight history category. This could indicate that other factors outside of physical activity participation may have a greater influence on weight loss maintenance.

When comparing physical activity levels between PWD and PWoD in the overall sample, PWD reported more sedentary time and lower levels of physical activity across several domains, specifically with higher odds of reporting no physical activity at all. Additionally, when investigating only those in the “Successful” group, PWD still reported more sedentary time and higher odds of reporting no leisure physical activity than PWoD. Given that among those that were active, there were no differences in physical activity between PWD and PWoD, this could indicate that more PWD may have difficulty beginning engagement in physical activity at all. The higher odds for PWD to report no physical activity engagement when compared to PWoD in this sample may be due to environmental barriers experienced by this population,^16,17^ limiting opportunities for engagement. Barriers include a lack of transportation or accessible facilities, or lack of staff knowledgeable in PA for PWD,.^16,17^ In addition to these environmental constraints, individual-level factors such as disability type and degree of functional limitation may also influence physical activity engagement.^18^ Evidence suggests that those with more self-reported limitations related to their disability may also participate in less physical activity.^18^ It is important to develop accessible programs and interventions to promote physical activity and reduce sedentary time in PWD with varying levels of functional limitation. Outside of weight loss and maintenance, physical activity is beneficial for overall health and reduced risk of chronic disease.^31^ Recently, guidelines were developed for encouraging physical activity engagement for PWD through ten target areas, including increased training, creation of accessible environments and programs, and policy changes.^32^ These guidelines should be taken into consideration in the development of physical activity programs for PWD to ensure programs are appropriate for all PWD, including those with more severe limitations.

Current literature has suggested that physical activity is a key component of weight loss maintenance for PWoD,^11-13^ however the present results indicate this may not be true for PWD. Among the present sample of PWD, none of the analyses comparing physical activity based on weight loss history were statistically significant. These findings may hold relevance and should be evaluated in the future using a larger sample and/or objective measures of physical activity. Evidence from other work has suggested that dietary strategies may be more important for weight loss in PWD,^33^ and this could be a greater influence in WLM as well. More research is needed to investigate factors associated with long-term weight management in PWD, and to develop effective, inclusive behavioral weight management programs for this population. In this sample, many PWD reported disabilities such as general joint pain and osteoarthritis, which can still pose meaningful barriers to physical activity. However, because these conditions may involve fewer functional limitations than other disability types, future analyses could restrict inclusion to individuals with more substantial mobility impairments or stratify results by disability severity. Our current sample size was not sufficient for these stratified analyses, but this should be considered in future work.

In this analysis, those who used assistive devices for ambulation reported more sedentary time, however, the hypothesis that those using assistive devices would report less total physical activity and leisure physical activity was not supported by the results. Given that assistive devices typically indicate greater functional limitations, which may influence physical activity participation,^17,34^ this result was unexpected. Although no statistical differences in physical activity were observed in this analysis, post-hoc power analyses indicated that this study had low power (10–39 %, depending on domain) to detect small effects when comparing PWD based on assistive device use. Therefore, these null findings should be interpreted with caution, and larger studies may be needed to clarify whether meaningful differences exist in this subgroup. Notably, the questionnaires used in this study did not ask about how often these assistive devices are used, which is an important consideration for future work. The most commonly reported assistive device was a cane, followed by a wheelchair and a walker, but it is unclear if these are used by participants intermittently or all of the time, which could influence physical activity engagement. Due to a limited sample size, this analysis did not compare physical activity engagement based on the type of assistive device (ex., wheelchair vs. cane), which could also present different limitations and barriers to physical activity. Future work should consider stratifying analyses by device type and usage patterns. As noted above, research has demonstrated that those with more severe limitations related to their disability may experience additional barriers to increased physical activity participation.^17,18,34^ Therefore, despite a lack of association found in this present analysis, emphasis should be placed on creating inclusive physical activity programs for persons with varying levels and severities of disability.

When comparing the overall sample of PWD versus PWoD, PWD reported more sedentary time than PWoD. Among PWD, those who used assistive devices also reported more sedentary time than PWD who did not use assistive devices. Independent of physical activity engagement, higher sedentary time has been associated with heart disease, diabetes, depression, and other chronic health conditions.^35^ Given that sedentary time was higher in PWD versus PWoD, PWD may be at particular risk for higher sedentary time, which may, in turn, influence overall health. In addition to creating accessible physical activity programs, importance should also be placed on reducing sedentary time or breaking up bouts of sedentary time with physical activity when possible for PWD.

A limitation of this study is the observational and self-report study design, which limits the types of conclusions that can be drawn. The scoring guidelines also indicate scoring any bouts of activity less than 10 min as 0 min.^23-25^ Recent evidence has supported shorter bouts of physical activity, including those less than 10 min, for health promotion.^36^ It has been reported that PWD may engage in shorter bouts of activity,^37^ and this may not be accurately captured using the current scoring guide for the IPAQ. As a result, the standard scoring methods may underestimate total physical activity in PWD. It is recommended that these results be confirmed using objective measurements of physical activity, including through accelerometers which can capture total physical activity counts. Additionally, many of the PWD in this sample reported joint pain or osteoarthritis as the disability or mobility-limiting condition, with fewer reporting other chronic conditions, such as spinal cord injury, paralysis, or multiple sclerosis, which could pose even more mobility-related limitations. This led to smaller sample sizes for these conditions thus, we were not able to compare based on disability type or severity. This could be an important consideration for the future with larger sample sizes. Also, this study was not able to evaluate the types of physical activity used during weight loss and weight loss maintenance efforts. Finally, a large number of participants were lost due to incomplete disability status information (n > 3000) which limited the sample size for analysis. The questions about disability status were not implemented in the original IWCR packet, leading to missing data. In the future, disability status questions should be included in the initial phase of questionnaires for participants during research studies in order to maximize sample size for similar analyses in the future.

PWD report less physical activity than PWoD, even among those who have achieved weight loss maintenance success. Programs and interventions should be designed to include PWD with varying levels of disability severity to promote physical activity engagement and reduce sedentary time. Evidence from this analysis indicates that physical activity may not be critical for PWD to maintain lost weight, and other strategies for weight maintenance in this population should be explored.

## Figures and Tables

**Table 1:** Participant characteristics.

Demographic	PWD (n = 193)	PWoD (n = 386)
Age, M (SD)^a^	57.5 (11.2)	52.6 (14.2)
BMI, M (SD)^a^	35.6 (10.7)	31.0 (7.4)
Female, n (%)	156 (80.8)	310 (80.3)
Race, n (%)		
American Indian or Alaska Native	0 (0.0)	1 (0.3)
Asian	1 (0.5)	8 (2.1)
Native Hawaiian or other Pacific Islander	0 (0.0)	1 (0.3)
Black or African American	31 (16.1)	52 (13.5)
White or Caucasian	146 (75.6)	311 (80.6)
More than one race	9 (4.7)	6 (1.6)
Other	1 (0.5)	2 (0.5)
Prefer not to specify	1 (0.5)	4 (1.0)
Unknown	1 (0.5)	0 (0.0)
Did not report	3 (1.6)	1 (0.3)
Ethnicity, n (%) Hispanic	8 (4.1)	18 (4.7)
Non Hispanic	173 (90.0)	359 (93.0)
Prefer not to specify	3 (1.6)	6 (1.6)
Unknown	3 (1.6)	0 (0.0)
Did not report	6 (3.1)	3 (0.8)
Income, n (%)^a^		
Less than $25,000	43 (22.3)	25 (6.5)
$25,000-$49,999	44 (22.8)	51 (13.2)
$50,000-$79,99	37 (19.2)	96 (24.9)
$80,000-$130,000	40 (20.7)	111 (28.8)
Greater than $130,000	22 (11.4)	98 (25.4)
Did not Report	7 (3.6)	5 (1.3)
Highest Level of Education, n (%)^a^		
High School Diploma or GED	18 (9.3)	13 (3.4)
Some college/Associates degree	58 (30.0)	60 (15.5)
College degree	59 (30.6)	140 (36.3)
Non-doctoral graduate degree	34 (17.6)	99 (25.6)
Doctoral degree	22 (11.4)	53 (13.7)
Did not report	2 (1.0)	21 (5.4)
Employment Status, n (%)^a^		
Full-time employment	52 (26.9)	232 (601)
Part-time employment	21 (10.9)	40 (10.3)
Unemployed, actively seeking employment	8 (4.1)	10 (2.6)
Not employed, not seeking employment	109 (56.5)	103 (26.7)
Did not report	3 (1.6)	1 (0.3)
Receives Food Stamps	8 (4.1)	11 (2.8)
Receives WIC Benefits	0 (0.0)	2 (0.5)
Receives Government Cash Assistance	5 (2.6)	5 (1.3)

Abbreviations: M, mean; PWD, people with physical disability; PWoD, people without physical disability; SE, standard error; WIC, Women, Infants, and Children.

a Indicates significant difference between PWD and PWoD.

**Table 2:** Disability and assistive device

Condition	N (% of PWD) (n=193)
Joint Pain	138 (71.5)
Osteoarthritis	96 (49.7)
Edema	36 (18.7)
Peripheral neuropathy	32 (16.6)
Multiple Sclerosis	25 (13.0)
Spinal Cord Injury	24 (12.4)
Paralysis	20 (10.4)
Stroke	13 (6.7)
Macular Degeneration	11 (5.7)
Traumatic Brain Injury	10 (5.2)
Rheumatoid Arthritis	10 (5.2)
Parkinson's	7 (3.6)
Muscular Dystrophy	6 (3.1)
Spina Bifida	5 (2.6)
Cerebral Palsy	4 (2.1)
Blind	4 (2.1)
Diabetic Retinopathy	4 (2.1)
Amputation	3 (1.6)
Postural orthostatic tachycardia syndrome	1 (0.5)
Other	77 (39.0)
Assistive Device Use	N (% of users) (n=81)
Cane	50 (61.7)
Walker	22 (27.2)
Push Wheelchair	22 (27.2)
Rollator	15 (18.5)
Power Wheelchair	15 (18.5)
Crutch	4 (4.9)
Knee Scooter	1 (1.2)
Guide dog	1 (1.2)
Other	7 (8.6)

**Table 3:** Physical activity across people in the “successful” group by disability status (n = 201).

Outcome	Effect Measure	Estimate	95 % CI	*p*-value
Sedentary Time (min/week)^a^	β	0.48	(0.15, 0.80)	0.004
Total Physical Activity (score)				
*Odds of zero activity*	OR	–	–	–
*Activity level (if non-zero)*	IRR	0.86	(0.64, 1.15)	0.309
Total Leisure PA (MET-min/week)				
*Odds of zero activity*	OR	4.25	(1.99, 9.05)	<0.001
*Activity level (if non-zero)*	IRR	1.14	(0.78, 1.67)	0.508
Moderate Leisure PA (MET-min/week)				
*Odds of zero activity*	OR	0.78	(0.37, 1.66)	0.516
*Activity level (if non-zero)*	IRR	1.17	(0.80, 1.72)	0.425
Vigorous Leisure PA (MET-min/week)				
*Odds of zero activity*	OR	1.70	(0.79, 3.67)	0.177
*Activity level (if non-zero)*	IRR	1.04	(0.73, 1.47)	0.832

*Note*. Results compare persons with disability (PWD) to persons without disability (PWoD, reference group). All models adjusted for age.

*Abbreviations*. CI, confidence interval; IRR, incidence rate ratio; MET, metabolic equivalent of task; min, minute; OR, odds ratio; PA; physical activity; .

b Model for zero total physical activity did not converge due to quasi-complete separation; see text for descriptive statistics.

a Square-root transformed for analysis.

**Table 4:** Physical activity across people in the “regain” group by disability status (n = 335).

Outcome	Effect Measure	Estimate	95 % CI	*p*- value
Sedentary Time (min/week)^a^	β	0.350	(0.13, 0.58)	0.002
Total Physical Activity (score)				
*Odds of zero activity*	OR	3.211	(1.28, 8.05)	0.013
*Activity level (if non-zero)*	IRR	0.936	(0.73, 1.19)	0.593
Total Leisure PA (MET-min/week)				
*Odds of zero activity*	OR	1.574	(0.99, 2.49)	0.054
*Activity level (if non-zero)*	IRR	1.078	(0.81, 1.44)	0.608
Moderate Leisure PA (MET-min/week)				
*Odds of zero activity*	OR	0.566	(0.31, 1.05)	0.070
*Activity level (if non-zero)*	IRR	1.009	(0.74, 1.38)	0.957
Vigorous Leisure PA (MET-min/week)				
*Odds of zero activity*	OR	1.625	(0.86, 3.08)	0.137
*Activity level (if non-zero)*	IRR	1.048	(0.68, 1.62)	0.834

*Note*. Results compare persons with disability (PWD) to persons without disability (PWoD, reference group). All models adjusted for age.

*Abbreviations*. CI, confidence interval; IRR, incidence rate ratio; MET, metabolic equivalent of task; OR, odds ratio; PA; physical activity; .

a Square-root transformed for analysis.
